# Implicit beliefs about ideal body image predict body image dissatisfaction

**DOI:** 10.3389/fpsyg.2015.01402

**Published:** 2015-10-08

**Authors:** Niclas Heider, Adriaan Spruyt, Jan De Houwer

**Affiliations:** Department of Experimental Clinical and Health Psychology, Ghent UniversityGhent, Belgium

**Keywords:** implicit attitudes, implicit beliefs, body image, body dissatisfaction, Implicit Relational Assessment Procedure, IRAP

## Abstract

We examined whether implicit measures of actual and ideal body image can be used to predict body dissatisfaction in young female adults. Participants completed two Implicit Relational Assessment Procedures (IRAPs) to examine their implicit beliefs concerning actual (e.g., I *am* thin) and desired ideal body image (e.g., I *want to be* thin). Body dissatisfaction was examined via self-report questionnaires and rating scales. As expected, differences in body dissatisfaction exerted a differential influence on the two IRAP scores. Specifically, the implicit belief that one is thin was lower in participants who exhibited a high degree of body dissatisfaction than in participants who exhibited a low degree of body dissatisfaction. In contrast, the implicit desire to be thin (i.e., thin ideal body image) was stronger in participants who exhibited a high level of body dissatisfaction than in participants who were less dissatisfied with their body. Adding further weight to the idea that both IRAP measures captured different underlying constructs, we also observed that they correlated differently with body mass index, explicit body dissatisfaction, and explicit measures of actual and ideal body image. More generally, these findings underscore the advantage of using implicit measures that incorporate relational information relative to implicit measures that allow for an assessment of associative relations only.

## Introduction

Body (image) dissatisfaction can be defined as the negative attitude toward one's own body resulting from a perceived discrepancy between the actual body image (i.e., perceptions, thoughts, and feelings concerning one's actual physical appearance; e.g., Cash, 1990) and the ideal body image (i.e., internalized ideals about one's physical appearance; e.g., Cooper and Taylor, 1988; Higgins, 1989; Williamson et al., 1990; Strauman et al., 1991; Williamson et al., 1993). Because body dissatisfaction plays a central role in the causation and maintenance of eating disorders (Stice, 2001; Fairburn and Harrison, 2003; American Psychiatric Association, 2013), behavioral scientists have long sought ways to measure the degree of dissatisfaction with one's personal physical appearance (e.g., Slade and Russell, 1973; Allebeck et al., 1976; Freeman et al., 1984; Schlundt and Johnson, 1990; Bessenoff and Sherman, 2000; Degner and Wentura, 2009; Roddy et al., 2010, 2011; Juarascio et al., 2011; Bluemke and Friese, 2012; Parling et al., 2012). Most often, they relied on the use of direct self-report measures (i.e., questionnaires), but it is well-known that such measures can be susceptible to social desirability and impression management (Cronbach, 1990; Holtgraves, 2004). In the context of eating disorders, for example, respondents may be motivated to respond untruthfully when completing an explicit measure of body dissatisfaction because they may be facing far-reaching therapeutic consequences (e.g., compulsory admission). In addition, self-report measures are, by definition, unsuited to capture attitudes that are introspectively unidentified (Greenwald and Banaji, 1995). Accordingly, behavioral scientists have begun developing diagnostic instruments that allow for an assessment of body dissatisfaction in an indirect way, that is, without having to ask for a direct self-assessment. Instead, inter-individual differences are inferred from a respondent's response pattern in well-controlled computer tasks, often referred to as *implicit measures* (De Houwer et al., 2009).

Hitherto, several attempts have been undertaken to develop implicit measures of body-related attitudes (e.g., Bessenoff and Sherman, 2000; Ahern et al., 2008; Watts et al., 2008; Degner and Wentura, 2009; Roddy et al., 2010, 2011; Juarascio et al., 2011; Bluemke and Friese, 2012; Parling et al., 2012). Consider, for example, the findings of Bluemke and Friese (2012). In their adaptation of the Implicit Association Test (IAT; Greenwald et al., 1998), stimuli referring to *thinness* and *overweight* and stimuli related to either *the self* or related to *a well-known other person* (i.e., a friend or a relative) were presented one by one on a computer screen. In a first critical block of trials, participants were asked to press one key as quickly as possible upon the presentation of a word referring to thinness (e.g., “skinny”) or a word related to the self (e.g., the participant's first name). The second key was to be pressed upon the presentation of a word referring to overweight (e.g., “fat”) or a word related to another person (e.g., the first name of a friend). In a second critical block, response assignments were reversed so that words referring to thinness and another person were assigned to the first key whereas words referring to overweight and the self were assigned to the second key. Based on the assumption that it is easier to respond when concepts assigned to the same key are associated in memory, a person's implicit body image was then inferred from the difference in performance between the two critical blocks. More specifically, participants with a thin body image were expected to perform best when words relating to the self and thinness were assigned to the same key whereas participants with an overweight body image were expected to perform best when words relating to the self and overweight were assigned to the same key. In sum, the IAT measure developed by Bluemke and Friese (2012) was designed to capture inter-individual differences in the strength of association between the concepts *self* and *body size* (i.e., thinness/overweight).

Body dissatisfaction, however, by definition comprises more than a simple association between the self and body size because it is driven by the (perceived) discrepancy between one's ideal and actual body image. Crucially, both ideal and actual body image involve a relation between the concepts *self* and *body size* but differ with regard to how those concepts are related. More specifically, beliefs about actual body image are characterized by a descriptive relation (e.g., I *am* thin). In contrast, beliefs about ideal body image relate the self to body size in terms of desirability (e.g., I *want to be* thin). Clearly, these two beliefs are fundamentally different, yet the IAT measure like the one developed by Bluemke and Friese (2012) is unable to differentiate between them as the IAT was designed to capture the associative, unqualified strength between two concepts.

As pointed out by Hughes et al. (2012; also see De Houwer, 2014), this is a property not only of the IAT but also of several other implicit measures such as the (standard) evaluative priming task (EPT; Fazio et al., 1995) and the affect misattribution paradigm (AMP; Payne et al., 2005)^1^. Within the tradition of Contextual Behavioral Science (Hayes et al., 2012) and Relational Frame Theory (RFT; Hayes et al., 2001), however, a new implicit measure has emerged that was designed specifically to capture inter-individual differences in the extent to which respondents relate stimuli in a specific manner. Known as the Implicit Relational Assessment Procedure (IRAP; Barnes-Holmes et al., 2006), this new implicit measure requires participants to respond to complex relational information in a manner that is either in line or at odds with their prior learning history. Crucially, task performance in the IRAP is assumed to depend on the extent to which the response rules coincide with an individual's earlier learning experiences. Accordingly, by examining which relational information results in optimal task performance given a specific response rule, one can learn about the precise way in which respondents have learned to relate specific stimuli (i.e., in RFT terminology, about their *brief and immediate relational response*). Translated to cognitive-psychological terms, one could also say that the IRAP capitalizes on the assumption that it is simply easier to respond in a manner that is consistent with one's personal beliefs than it is to respond in a manner that is inconsistent with one's personal beliefs. Crucially, the IRAP allows for an assessment of how people relate concepts under conditions of automaticity. It could thus be hypothesized that the IRAP has the potential to outperform classic implicit measures such as the EPT and the IAT whenever relational information is critical.

As an example, consider the studies of Remue and colleagues who examined ideal and actual self-esteem in a sample of dysphoric students and a non-dysphoric control group (Remue et al., 2013, 2014). To capture ideal self-esteem, participants were presented, on each of a series of trials, either with the target stimulus “I want to be” or “I do not want to be” together with either a positive or negative adjective. Each combination of both stimuli was thus either congruent or incongruent with having positive ideal self-esteem (e.g., “I want to be + good” and “I want to be + bad”, respectively). In one block of trials, participants were asked to respond as if they possessed positive ideal self-esteem. They were thus required to select the response “true” whenever a stimulus combination was presented that referred to having positive ideal self-esteem (e.g., “I want to be + good”). Conversely, they were required to select the response “false” whenever a stimulus combination was presented that referred to having negative ideal self-esteem (e.g., “I want to be + bad”). In a second block of trials, participants were asked to respond as if they possessed negative ideal self-esteem. Accordingly, they were expected to select the response “true” whenever a stimulus combination was presented that referred to having negative ideal self-esteem (e.g., “I want to be + bad”). Whenever, a stimulus combination was presented that referred to having positive ideal self-esteem (e.g., “I want to be + good”), they were expected to select the response “false”. As argued above, the IRAP is based on the assumption that it is easier to respond in a manner that is consistent with one's personal beliefs than it is to respond in a manner that is inconsistent with one's personal beliefs. A person's level of ideal self-esteem was thus inferred from the difference in performance between the two critical blocks. More specifically, participants with positive ideal self-esteem were expected to perform best when responding to stimulus combinations in line with possessing positive ideal self-esteem. Participants with negative ideal self-esteem, in contrast, were expected to perform best when responding to statements in line with possessing negative ideal self-esteem. It was anticipated that the desire to be good (i.e., positive ideal self-esteem) would be more pronounced in dysphoric students than in non-dysphoric controls, as was indeed observed by Remue et al. (2013, but see Remue et al., 2014).

Crucially, Remue and colleagues also administered a second IRAP that was designed to capture actual self-esteem. This second IRAP was identical to the first one, except for the fact that participants were now (a) presented with stimulus combinations that referred to actual self-esteem (e.g., “I am + good”) and (b) were required to respond as if they did or did not possess positive self-esteem. Despite the structural similarity between the two IRAPs, the results obtained with the second IRAP revealed no (Remue et al., 2014) or even a reversed difference between dysphoric and non-dysphoric students (i.e., more positive actual self-esteem in non-dysphoric than in dysphoric students; Remue et al., 2013). These findings are in line with the notion that ideal and actual self-esteem are two different constructs and should therefore be measured independently from each other. More generally, because the essential difference between ideal and actual self-esteem concerns the quality of the relation between the self and positive/negative affect, these findings underscore the need for implicit measures that are sensitive to relational information.

Accordingly, the aim of the present research was to develop an implicit measure of body dissatisfaction that takes into account the way in which the concepts self and body-size are related. To that end, participants were asked to perform two IRAPs in quick succession, one to capture the extent to which participants endorsed or rejected beliefs reflecting their actual body image (e.g., “I am thin”, “I am not overweight”) and one to capture the extent to which participants endorsed or rejected beliefs reflecting their ideal body image (e.g., “I want to be thin”, “I don't want to be overweight”). Based on the definition of body dissatisfaction as the negative attitude toward one's own body that results from the (perceived) discrepancy between actual and ideal body image, we expected the scores of the actual and ideal body image IRAPs to depend upon the degree of self-reported body dissatisfaction. More specifically, given that adults typically strive to be thin rather than overweight (i.e., thin-ideal internalization; e.g., Thompson and Stice, 2001), we expected the belief to be thin to be more pronounced in those participants low in body dissatisfaction as compared to participants high in body dissatisfaction. In contrast, we expected that the desire to be thin would be less pronounced in those participants low in body dissatisfaction as compared to participants high in body dissatisfaction. It may be noted that all participants were (female) university students who were tested anonymously in a non-clinical context. It therefore seems unlikely that they would be unwilling to respond in a truthful manner when completing the explicit measures. Accordingly, we also expected implicit and explicit measures of actual and ideal body image to be correlated in this particular sample.

## Methods

### Ethics statement

Participants gave written informed consent prior to their participation and received course credit in exchange for their participation. The experiment was approved by the ethics committee of the Faculty of Psychology and Educational Sciences at Ghent University.

### Participants

Between 3 and 4 weeks prior to the actual experiment, 307 students at Ghent University completed the body dissatisfaction subscale of the Eating Disorders Inventory (EDI; Garner et al., 1983) during an online screening study that involved several questionnaires. To ensure the inclusion of participants that were either highly satisfied or highly dissatisfied with their body, we invited all female students who had scored within the first and forth quartile of the total EDI distribution to participate in an individual lab session (*N* = 112). In total, 52 female students (*M* = 19.6 years, *SD* = 4.8) responded to our invitation and participated in the actual laboratory experiment. Four of these participants failed to complete at least one of the two IRAP measures and were therefore excluded. All participants were Dutch speakers and had normal or corrected-to-normal vision.

### Measures

#### Self-report measures

Body dissatisfaction was assessed by means of the body dissatisfaction subscale of the Eating Disorder Inventory (EDI, 9 items; Garner et al., 1983) as well as the body dissatisfaction subscale of the Body Attitude Test (BAT, 7 items; Probst et al., 1995). Both measures have excellent psychometric qualities (e.g., Probst et al., 1997; Clausen et al., 2011; Vanderlinden et al., 2012). Actual and ideal body image were measured using the female version of the Contour Drawing Rating Scale (CDRS; Thompson and Gray, 1995). The CDRS consists of nine schematic (female) figures of varying sizes ranging from *underweight* (1) to *overweight* (9). Participants completed the CDRS twice, once with the request to indicate their actual body image and once with the request to indicate their ideal body image. Finally, we computed two Body Mass Indices (BMI) for each participant, once using their self-reported weight and height (i.e., self-reported BMI) and once using their factual weight (i.e., factual BMI). Discrepancies between self-reported BMI and factual BMI were unrelated to all other (indirect and direct) measures. Accordingly, only the factual BMI data were used. Both indexes correlated highly, *r* = 0.96.

#### IRAPs

Participants completed two IRAPs, one to capture actual body image (i.e., actual-IRAP) and one to capture ideal body image (i.e., ideal-IRAP). To capture actual body image, participants were presented with combinations of the stimuli “I am” or “I am not” (in the IRAP literature referred to as *sample stimuli*) and one of 12 words referring to the concepts thinness and overweight (in the IRAP literature referred to as *target stimuli*). The combination of sample and target stimuli resulted in 24 combinations. Twelve combinations were in line with the belief “I am thin” (e.g., “I am + slim”, “I am not + chubby”), and 12 combinations were in line with the belief “I am overweight” (e.g., “I am + chubby”, “I am not + slim”). Similarly, to capture ideal body image, the same set of 12 target stimuli was combined with the sample stimuli “I want to be” or “I don't want to be”. The resulting 24 combinations were thus either in line with the belief “I want to be thin” (e.g., “I want to be + slim”, “I don't want to be + chubby”) or in line with the belief “I want to be overweight” (“I want to be + chubby”, “I don't want to be + slim”). All target stimuli are presented in Table A1.

Both IRAPs consisted of six blocks of trials in which each of 24 combinations of sample and target stimuli was presented exactly once in random order (144 trials in total). Participants were asked to respond as fast as possible by pressing one of two response keys (i.e., the keys D and K). One of the keys indicated “true” whereas the other indicated “false”. Response assignments varied randomly from trial to trial. Accordingly, response assignments for each trial were signaled by the words “true” and “false” presented at the bottom left and the bottom right corner of the computer screen. To capture actual body F image, participants were asked to respond in line with the belief “I am thin” in one type of block (i.e., congruent block). In the second type of block (i.e., incongruent block), they were asked to respond in line with the belief “I am overweight”. Likewise, to capture ideal body image, participants were asked to respond in line with the belief “I want to be thin” in the congruent block. In the incongruent block, they were asked to respond in line with the belief “I want to be overweight”. Congruent and incongruent blocks were presented in an alternating order and all participants started with a congruent block of trials.

Each IRAP was preceded by a practice phase. All participants completed a congruent practice block followed by an incongruent practice block in which each of the 24 stimulus combinations was presented in a random order. Participants were instructed verbally to focus on response accuracy first and then to increase their response speed. Unless participants achieved an accuracy of more than 80% and a median response latency of less than 2000 ms in both practice blocks, a second pair of practice blocks was presented. If necessary, this procedure was repeated after the second pair of practice trials. If participants failed to reach the threshold criteria during the third pair of practice trials, the IRAP was stopped. This was the case for four participants who did not complete at least one of the IRAPs.

Each trial was started with a 400-ms blank white screen. Afterwards, one of the 24 stimulus combinations of a sample and a target stimulus was presented in two rows at the center of the upper half of the computer screen in black color, Arial font size 26. The response assignments for each trial (i.e., the words “true” and “false”) were presented in a green color, Arial font size 36, at the bottom left and the bottom right corner of the computer screen. In case of an incorrect response, a red X was presented in Arial font size 48 below the stimulus combination. Participants were required to correct an erroneous response in order to proceed to the next trial. After each block, participants were presented with feedback about their accuracy (in percentages) and speed of responding (in median response latencies), based on the last 24 trials, in red color, Arial font size 12. Each block of trials was preceded by the presentation of a reminder about the overall response rule, Arial font size 14 (e.g., “Please respond as if you are thin and are not overweight”). All stimuli were presented on a 19-inch VGA screen (75 Hz, 1024 × 768 pixels) and implemented using the 2012 version of the IRAP software, downloaded from http://www.irapresearch.org.

### Procedure and group assignment

All participants were tested individually and each experimental session took approximately 45 min. Participants completed the two IRAPs in a counterbalanced order. They then completed the EDI, the BAT, and the CDRSs for actual and ideal body image, in this specific order. Finally, the information needed for the calculation of the BMI indices was registered.

Because participant sampling was conducted anonymously via an online recruitment system, group assignment was based on the explicit measures of body dissatisfaction collected during the actual lab session. The EDI and BAT scores were highly correlated in our sample, *r* = 0.91, and were therefore aggregated. More specifically, both scores were first standardized across participants and then averaged for each individual. Based on this (sum) score of body dissatisfaction, participants were assigned to either the low or the high body dissatisfaction group by means of a cluster analysis. In the low body dissatisfaction group (*n* = 24), the mean (standardized) score was −0.90 (*SD* = 0.27, *min* = −1.17, *max* = −0.03). In the high body dissatisfaction group (*n* = 24), the mean (standardized) score was 0.90 (*SD* = 0.43, *min* = 0.04, *max* = 1.75). There was no overlap between both groups.

## Results

### Data preparation

For each participant and each version of the IRAP, the raw response latencies of the six experimental blocks were aggregated in a single overall D-score using the algorithm described by Greenwald et al. (2003). In a first step we excluded all response latencies above 10,000 ms (0.10%). Other criteria for data exclusion as specified in the scoring algorithm (e.g., responses faster than 300 ms on more than 10% of the trials) were not met. We then calculated standard deviations on the basis of all response latencies observed in subsequent pairs of congruent and incongruent blocks (i.e., Block 1 & 2, 3 & 4, and 5 & 6). Next, mean response latencies were calculated for each of the six blocks. Three difference scores were then calculated, one for each pair of congruent and incongruent blocks, by subtracting the mean response latency observed in the congruent block from the mean response latency observed in the incongruent block. Each difference score was divided by its corresponding standard deviation, yielding three D-scores, one for each pair of blocks. Finally, the three D-scores were averaged to obtain one overall D-score^2^. D-scores were calculated so that positive values indicate a higher degree of a thin body image belief (actual or ideal).

### Effects at the group level

**Figure 1:**
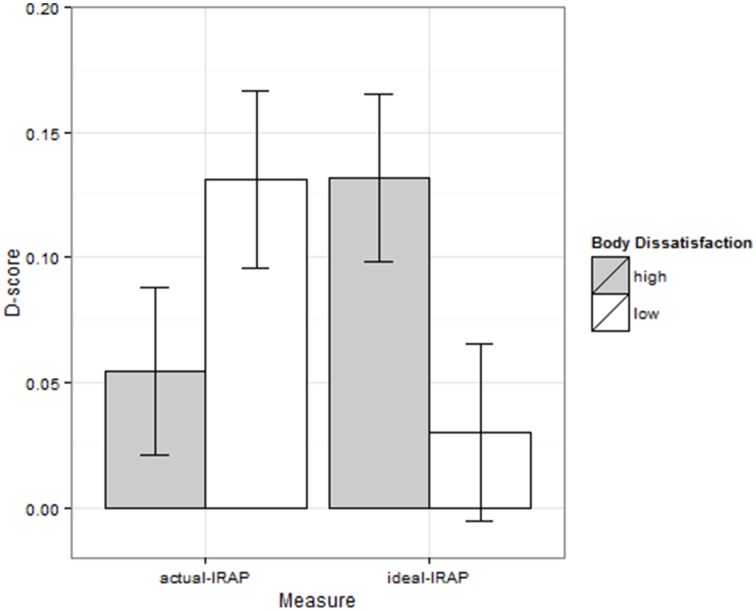
**D-scores of actual and ideal body image IRAP as a function of group membership**.

To investigate whether implicit measures of actual and ideal body image were dependent upon the degree of self-reported body dissatisfaction, a 2 (body dissatisfaction: high vs. low) by 2 (IRAP: actual vs. ideal) ANOVA was conducted^3^. As expected, we found a significant interaction between body dissatisfaction and IRAP, *F*_(1, 46)_ = 6.71, *p* = 0.013, η${}_{\rho }^{2}=0.13$, indicating that the two groups responded differently to the two IRAPs. Participants low in body dissatisfaction scored higher on the actual-IRAP than participants high in body dissatisfaction, 0.13 (*SD* = 0.17) vs. 0.05 (*SD* = 0.16), respectively. In contrast, participants high in body dissatisfaction scored higher on the ideal-IRAPthan participants low in body dissatisfaction, 0.13 (*SD* = 0.16) vs. 0.03 (*SD* = 0.17), respectively (see Figure 1). This pattern of results is consistent with our hypotheses (a) that the implicit belief that one is thin is more pronounced in individuals who are low in body dissatisfaction as compared to individuals who exhibit a high degree of body dissatisfaction, and (b) that the implicit desire to be thin is more pronounced in individuals who are high in body dissatisfaction as compared to individuals who exhibit a low degree of body dissatisfaction. Additional *t*-tests showed, however, that the D-scores of the actual-IRAP did not differ significantly between groups, *t*_(46)_ = 1.57, *p* = 0.122, *d* = 0.45. In contrast, the D-scores of the ideal-IRAP did differ significantly between the groups, *t*_(46)_ = 2.12, *p* = 0.040, *d* = 0.61. Differences between actual and ideal body image were significant for participants low in body dissatisfaction, *t*_(46)_ = 2.02, *p* = 0.049, *d* = 0.58, and marginally significant for participants high in body dissatisfaction, *t*_(46)_ = 1.65, *p* = 0.105, *d* = 0.48. The ANOVA did not reveal other significant effects, all *F*s < 1, all *p*s > 0.712.

### Correlational analyses

For exploratory reasons, we also computed all pairwise correlations between the IRAP scores, the CDRS measures, the explicit measure of body dissatisfaction, and the BMI (see Table 1). Note, however, that most variables were not normally distributed because our sample consisted of participants who exhibited either a high or low degree of body dissatisfaction. Accordingly, Spearman's rank order correlation coefficients were computed rather than Pearson product-moment correlation coefficients. In line with the results presented above, the ideal-IRAP correlated or tended to correlate positively with the actual-CDRS, the ideal-CDRS, the explicit measure of body dissatisfaction, and the BMI. Conversely, the actual-IRAP correlated negatively with each of these measures, albeit not significantly so. Adding further weight to the idea that both IRAP measures captured different underlying constructs, the degree to which the two IRAP measures correlated with each of the other variables was reliably different for the actual-CDRS, the ideal-CDRS, and the explicit measure of body dissatisfaction, *t*s < 2.01, *p*s < 0.05. For the BMI, the difference between the correlation with the ideal-IRAP and the actual-IRAP just missed significance, *t*_(46)_ = 1.91, *p* = 0.06.

**Table 1:** **Descriptive statistics of and Spearman's rank order correlations between measures**.

		***M***	***SD***	**min**	**max**	**1**	**2**	**3**	**4**	**5**	**6**
1	Actual-IRAP	0.12	0.18	−0.31	0.59	-	−0.08	−0.24^+^	−0.27^+^	−0.12	−0.12
2	Ideal-IRAP	0.10	0.17	−0.35	0.39		-	0.39^**^	0.27^+^	0.30^*^	0.28^*^
3	Actual-CDRS	5.10	1.93	2	9			-	0.60^***^	0.82^***^	0.77^***^
4	Ideal-CDRS	3.60	1.12	1	6				-	0.17	0.63^***^
5	EBD	0	0.98	−1.71	1.74					-	0.61^***^
6	BMI	21.90	3.37	17.39	32.20						-

*IRAP, Implicit Relational Assessment Procedure; CDRS, Contour Drawing Rating Scale; EBD, explicit body dissatisfaction, based on averaged scores of the body dissatisfaction subscales of the Eating Disorders Inventory (EDI) and the Body Attitude Test (BAT); BMI, Body Mass Index*.

+ *p < 0.10*,

* *p < 0.05*,

** *p < 0.01*,

*** *p < 0.001*.

### Hierarchical regression analyses

To examine whether the IRAP scores can be used to predict body dissatisfaction over and above self-report measures of actual and ideal body image, a hierarchical regression analysis was performed. Specifically, we compared a model in which body dissatisfaction was predicted by actual and ideal body image CDRS with a model that also included the two IRAP scores. Both actual and ideal body image CDRS predicted body dissatisfaction to a significant extent, *F*_(1, 45)_ = 254.76, *p* < 0.001, and *F*_(1, 45)_ = 65.93, *p* < 0.001, respectively. The model including the D-scores of both IRAPs did not, however, explain significantly more variance, *F* < 1.

### Reliability

For the EDI and BAT subscales of body dissatisfaction, Cronbach's alpha was 0.99 and 0.93, respectively. Test-retest reliability of the actual body image CDRS was *r* = 0.78, as reported by Thompson and Gray (1995). To obtain a measure of reliability for the IRAP measures, we first split each individual data set in two random halves. Next, two IRAP scores were calculated, one for each half, as well as the correlation (across participants) between these two IRAP scores. Finally, this process was repeated 100 times and the mean correlation was used as a measure of reliability. This procedure resulted in spearman-brown corrected mean split-half correlations of *Rsb* = 0.32 and *Rsb* = 0.42, for the actual and ideal body image IRAP, respectively.

## Discussion

The degree to which people are dissatisfied with their own body is a function of the (perceived) discrepancy between one's actual and ideal body image (e.g., Cooper and Taylor, 1988; Williamson et al., 1990, 1993; Strauman et al., 1991). We hypothesized that participants high and low in body dissatisfaction would differ not only in their self-reported degree of body dissatisfaction but also in their implicit beliefs concerning their actual and ideal body image. More specifically, we expected the implicit belief that one is thin to be more pronounced for participants low in body dissatisfaction as compared to participants high in body dissatisfaction. In contrast, we expected the implicit desire to be thin to be more pronounced in participants who exhibit a high degree of body dissatisfaction as compared to participants low in body dissatisfaction. Using the IRAP (Barnes-Holmes et al., 2006) as an implicit measure of beliefs, we found strong supporting evidence for both predictions. In addition, the pattern of correlations between each of the two IRAP measures and a number of other variables was quite different. In line with the idea that the desire to be thin must be higher in individuals who estimate their own physical appearance to be overweight, the ideal-IRAP correlated positively with the actual-CDRS. Likewise, significant positive correlations were observed with the BMI and explicitly measured body dissatisfaction. In contrast all correlations between the actual-IRAP scores and each of these measures were negative, albeit not significantly so. Taken together, these findings strongly suggest that both IRAP measures, despite their structural similarity, captured different underlying constructs.

These observations are important for two reasons. First, as pointed out above, an important rationale for using implicit measures is their alleged resistance to social desirability concerns and impression management. In addition, it has been argued that implicit measures may be used to capture traces of prior experience that are introspectively unidentified. It can thus be hypothesized that the added value of using implicit measures of actual and ideal body image to predict behavioral outcomes will be most pronounced when participants are somehow unwilling or unable to complete explicit measures of body dissatisfaction in a truthful manner. In this respect, it seems particularly interesting to use the IRAP measures developed here in the context of eating disorders (e.g., anorexia nervosa), for two reasons. First, patients suffering from eating disorder might be inclined to fake self-report measures of (ideal and actual) body image because of significant therapeutic consequences (e.g., compulsory admission). In addition, the discrepancy between actual body weight (BMI) and implicit beliefs about one's actual body weight might be an important cognitive marker for future therapeutic outcomes. In sum, while the current study revealed no added value of the IRAP measures over and above explicit measures (e.g., the CDRS measure of actual body image) in predicting self-reported body dissatisfaction, there are good reasons to suspect that implicit measures of ideal and actual body image may be much more instrumental in applied research contexts. Follow-up studies are needed, however, to verify whether the IRAP measures of body image can indeed predict behavioral outcomes (e.g., eating behavior, probability of relapse) over and above explicit measures.

A second reason why our findings are important concerns the use of implicit measures in general. For two decades now, implicit measures have been widely used in various research domains, including health and clinical psychology (e.g., Wiers et al., 2002, 2010; Stacy and Wiers, 2010; Teachman et al., 2010; Spruyt et al., 2013; Descheemaeker et al., 2014), forensic psychology (e.g., Snowden and Gray, 2010), and consumer psychology (e.g., Perkins and Forehand, 2010). Crucially, traditional implicit measures such as the IAT, EPT, and AMP were designed to capture the extent to which certain concepts are associated in memory (Hughes et al., 2012). Each of these measures, for example, can be readily used to capture the extent to which a certain class of stimuli (e.g., spiders) is associated with a positive or negative valence. In many cases, however, it is not only important to examine whether two concepts are related in memory but also the precise way in which they are related. As demonstrated by Remue et al. (2013, 2014), for example, it makes a tremendous differences to know whether someone has the implicit belief to be a person who actually *is* good or *wants to be* good. Likewise, the findings reported here demonstrate that it is important to distinguish between the implicit *desire to be* thin vs. the implicit *belief that one is* thin. Accordingly, it seems most interesting or even essential to invest in the development of implicit measures that are capable of tapping into relational information that is more complex than simple, unqualified associations (e.g., The Relational Responding Task, recently introduced by De Houwer et al., 2015).

To sum up, we used two IRAP measures: One to capture implicit beliefs concerning one's actual body image and one to capture implicit beliefs concerning one's ideal body image. Both IRAP measures were related to different outcome variables to a different extent, thereby underscoring the validity of both measures. More generally, our findings indicate that it is key to examine not only whether two concepts are related in memory but also *how* they are related. Future research concerning the applied value of implicit measures would thus benefit greatly from taking into account complex relational information.

### Conflict of interest statement

The authors declare that the research was conducted in the absence of any commercial or financial relationships that could be construed as a potential conflict of interest.

## Footnotes

^1^It might be noted that the priming paradigm can be adapted so as to allow for the measurement of (automatic) relational processing. For an example, see Heider et al. (Submitted).

^2^It may be noted that this approach diverges from the analysis strategy adopted by Barnes-Holmes et al. (2010). Instead of calculating a single, overall D-score on the basis of all trials, these authors suggested to calculate four different D-scores, one for each of type of trial (i.e., “I am + thin”, “I am not + thin”, “I am + overweight”, “I am not + overweight”, in case of the actual-IRAP). An overall D-score can then be obtained by averaging the four trial-specific D-scores. The rationale to depart from this approach is twofold. First, all four trial types are assumed the probe the exact same belief (within the same IRAP). Second, by aggregating all observations into a single D-score, the impact of outlying observations is much better controlled for. It may be noted that our findings are not contingent upon the inclusion or exclusion of trial type as a factor.

^3^The order in which the two IRAP tasks were completed was counterbalanced across participants. Reassuringly, our critical effect (i.e., the interaction between explicit body dissatisfaction and the type of IRAP) was not moderated by this counterbalancing factor, *F*_(1, 44)_ = 1.38, *p* = 0.247, $\eta_{\rho }^{2}=0.03$, nor were any other effects, all Fs < 1, all *ps* > 0.49. We therefore excluded the order factor from the analyses.

## Appendix

**Table A1:** **Target stimuli of the IRAP measures and English translations**.

**Thinness**	**Thickness**	**Thinness (English)**	**Thickness (English)**
Dun	Vet	Thin	Fat
Mager	Dik	Lean	Thick
Fijngebouwd	Zwaarlijvig	Fine boned	Obese
Smal	Gezet	Narrow	Squat
Tenger	Volslank	Slender	Curvaceous
Slank	Mollig	Slim	Chubby
